# Knowledge and use of thoracic manipulations by manual therapists in Belgium and the Netherlands

**DOI:** 10.1080/10669817.2025.2559023

**Published:** 2025-10-14

**Authors:** Andrei Sokolov, Benoît Beyer, Emiel Van Trijffel, Aldo Scafoglieri

**Affiliations:** a Faculty of Physical Education and Physiotherapy, University of Brussels (VUB), Brussels, Belgium; b Faculty of Human Movement Sciences, University of Brussels (ULB), Brussels, Belgium; c ZGT Academy, Hospital Group Twente, Almelo, NL, The Netherlands

**Keywords:** Thoracic spine, Manipulation, manual therapy, Survey, clinical practice, clinical reasoning

## Abstract

**Introduction:**

Beneficial effects of thoracic manipulations (TM) have been demonstrated; however, there is a lack of clarity regarding their use in clinical practice.

**Purpose:**

To investigate the knowledge and use of TM in primary care manual therapy in Belgium and the Netherlands.

**Methods:**

An online survey hosted on Google Forms was designed and distributed across various platforms to manual therapists and students enrolled in a Master’s/Postgraduate program in manual therapy. Data were collected regarding demographics, types of complaints, types of manipulation, and types of intervention. Participation occurred between 1 November 2022 and 31 July 2024.

**Results:**

One hundred and seven surveys were analyzed. Thoracic complaints treated with TM were mostly muscular in origin (94%). Most participants (79%) did not utilize a specific screening tool, instead relying on clinical experience to exclude red flags. The majority agreed that metabolic bone diseases and metastatic diseases (*n* = 92) are contraindications and that thoracic fractures (*n* = 77) are a risk factor for TM. Almost half (44%) did not differentiate between the upper and lower thoracic spine. TM for other regions, mostly for the cervical spine (94%), was considered. TM was preferred for its biomechanical effects, followed by neurophysiological and placebo effects.

**Conclusion:**

Most clinicians rely on experience rather than a pre-screening tool when assessing the thoracic region, focussing on excluding red flags and determining indications for TM, with biomechanical effects being the most favored rationale. Although most findings showed agreement, some inconsistencies were noted regarding screening and rationale, highlighting the importance of clinical reasoning for safe TM practice.

## Introduction

Manipulation of the spine, defined as a ‘separation (gapping) of opposing articular surfaces of a synovial joint, caused by a force applied perpendicularly to those articular surfaces, resulting in cavitation within the synovial fluid of that joint,’ is a commonly applied treatment for spinal complaints based on the various neurophysiological effects the intervention might have [1,2]. When a high-velocity low-amplitude (HVLA) force or thrust is applied to spinal joints, an audible ‘pop’ or ‘click’ may occur indicating cavitation of the joint, differentiating the technique from a regular mobilization [3].

Kinematic relationships between the thoracic spine and the neck and shoulder regions have been demonstrated [4,5]. Multiple studies have investigated the effectiveness of thoracic manipulations (TM) for complaints in these regions [6–11]. These studies suggest that TM may also be beneficial for regions adjacent to the thoracic spine.

In addition to positive findings reported in the research, adverse events (AEs) have also been documented, affecting the spinal cord, viscera, vertebrae, and vascular tissues [12]. Although the number of reported cases is limited, safe practice remains essential to minimize these risks. Because each AE is one too many, thorough screening, sound clinical reasoning, and a strong theoretical as well as practical background are required. However, concerns arise when patients are not screened for potential risks before manipulation. Evidence from the United States (US) and the United Kingdom (UK) indicates that only 9% (US) and 40% (UK) of therapists perform pre-manipulative screening for the thoracic spine [13,14].

Despite efforts by the International Federation of Manual and Musculoskeletal Physical Therapists (IFOMPT), which have resulted in a framework for the cervical spine [15] and various screening tools for the lumbar region [16,17], to our knowledge, no such framework exists for the thoracic spine. Although studies addressing safety and pre-manipulative screening exist, research on this topic remains limited [3,12–14].

The purpose of this was tofirst explore and then describe aspects of clinical reasoning related to the use of thoracic manipulations in manual therapy practice within the Belgian and Dutch contexts. Previous research examined knowledge of thoracic thrust joint manipulation (TTJM) and pre-TTJM examination across IFOMPT Member Organizations (MOs) and Registered Interest Groups (RIGs). Those findings revealed inconsistencies regarding knowledge of contraindications and precautions, beliefs about biomechanical effects, and highlighted the importance of clinical reasoning [12]. As this study continues that previous work, those findings were taken into consideration.

## Design and methods

### Ethics

The study was approved by the Review Board of the Advanced Master/Postgraduate program in Manual Therapy at the University of Brussels (VUB). Participant identities were not revealed during or after the study, as confidentiality was ensured.

### Design

An online survey consisting of 30 questions was hosted on Google Forms. The survey was written in Dutch, as it targeted therapists from Belgium and the Netherlands. The Checklist for Reporting of Survey Studies (CROSS) was followed [18]. Participation was openfrom 1 November 2022 to 31 July 2024. The survey could be accessed on any internet-connected electronic device.

### Survey description

The survey consisted of both open – and closed-ended questions, totaling 30 items, with an estimated completion time of 15–20 minutes. Some closed questions used agreement scales, while others did not. Participants were allowed to modify their responses before submission; however, all questions were mandatory, resulting in a complete dataset with no missing values. Participation was voluntary and limited to a single submission. The first nine questions collected demographic information about the therapists, including date of birth, gender, years licensed as a physiotherapist, work setting, years licensed as a manual therapist, location of TM training, number of hours of additional training related to the thoracic spine, years of experience in a musculoskeletal setting, and number of patients with spinal dysfunctions treated weekly. Questions 10 to 13 focused on thoracic complaints, including the types of complaints encountered, whether specific questions were asked when patients presented with thoracic symptoms, what tests were used, and whether other spinal regions were routinely assessed. In question 12, participants were asked to rate how often they used each listed test during thoracic examination, with response options ranging from ‘always’ to ‘no answer.’ Questions 14 to 24 addressed TM, including frequency of use, screening, contraindications, considerations, red flags, risk factors, differentiation between upper and lower thoracic regions, clinical reasoning, and technical preferences. Questions 17 to 20 specifically addressed contraindications, considerations, red flags, and risk factors. For these, participants indicated their level of agreement with each item on a scale from ‘strongly disagree’ to ‘strongly agree.’ In question 23, participants rated the perceived effects of TM using a scale from ‘most’ to ‘least.’ In question 24, participants indicated their preferred patient positioning for TM, ranging from ‘most utilized’ to ‘least utilized.’ Lastly, questions 25 to 30 touched on the treatment, the use of thoracic spine techniques for managing complaints in other body regions, and the types of thoracic spine interventions used for those complaints.

### Sample and recruitment

To be eligible, participants had to be licensed manual therapists (holding an official ‘manual therapist’ title) or manual therapy students (enrolled in an Advanced Master’s or Postgraduate program). Recruitment was conducted through multiple channels. Monthly invitations in the form of newsletters were emailed to all manual therapists listed in the databases of the Manual Therapy Association Belgium (MATHERA, 4263 members) and the Dutch Association for Manual Therapy (NVMT, 1386 members). Additionally, other forms of outreach were used, such as direct contact with universities, Facebook, LinkedIn, phone calls, a podcast, and in person interactions.

### Data analysis

Data was collected via Google Forms, which provided three options for analysis: 1) overview analysis; 2) analysis by question; and 3) analysis by survey. Answers to all questions were synthesized and incorporated. Excel spreadsheets were used to collect and import data, leading to the creation of figures to visualize the minimum, maximum, and percentages of the obtained results.

## Results

Of the manual therapists invited, 123 completed the survey. As all questions were mandatory, all surveys were fully completed before submission. During data analysis, 16 therapists had to be excluded as they did not meet the educational eligibility criteria. As such, the data analyzed are from 107 manual therapists and will be presented in four categories: 1) demographics; 2) thoracic complaints; 3) thoracic manipulations; and 4) treatment and intervention type.

### Demographics

Fifty-four males (50.5%) and 53 females (49.5%) completed the survey with ages ranging from 25 to 71 years. All were certified physiotherapists, with most having qualified between 6–10 years (33%). Additional interest was shown in the number of years the physiotherapists had their manual therapy certification with most certified less than 3 years (27%), followed by 4–6 years (25%), and over 16 years (23%). The majority worked in private practice (97%). Most received their training at the University of Brussels (VUB) (*n* = 36), followed by the University of Ghent (UG) (*n* = 30), and SOMT University of Physiotherapy (*n* = 22). Nearly half (41%) had less than 4 hours of additional training in the thoracic spine with most having worked between 6–10 years (32%) in a musculoskeletal setting. Lastly, regardless of the variety, the majority mentioned treating 6–9 patients weekly per spinal region.

### Thoracic complaints

**Figure 1.**
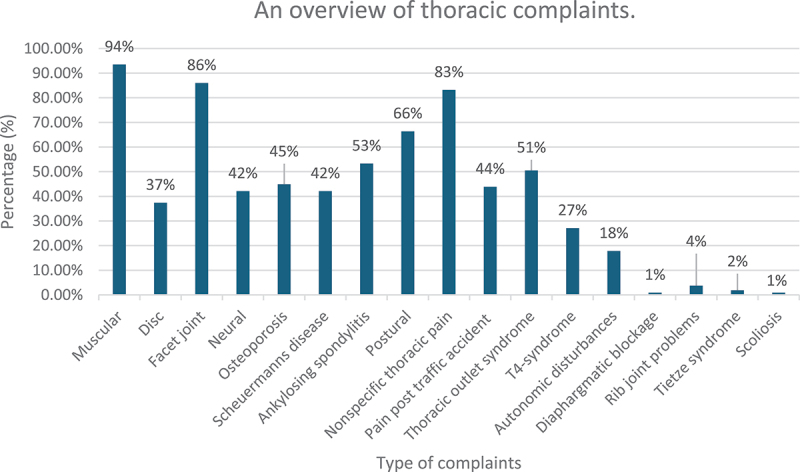
An overview of thoracic complaints.

A variety of thoracic complaints was seen by participating therapists with most complaints being muscular in origin (94%), related to facet joints (86%), or nonspecific thoracic pain (83%) (Figure 1). The more frequently asked questions were: 1) ‘pain with deep breathing?’ (97%); 2) ‘pain when coughing and sneezing?’ (94%); 3) ‘history of cancer?’ (84%).

As for testing, the most commonly used methods were palpation (*n* = 97), active range of motion (AROM) assessment (*n* = 90), and functional assessment (*n* = 82). In some cases, clinicians also utilized muscle testing (*n* = 84), Upper Limb Tension Test (ULTT) (*n* = 83), and special tests (*n* = 76). Regarding the assessment of the thoracic spine when treating other complaints, the following regions were most frequently considered: 1) cervical spine (95%); 2) shoulder (87%); and 3) lumbar spine (74%).

### Thoracic manipulations

**Figure 2.**
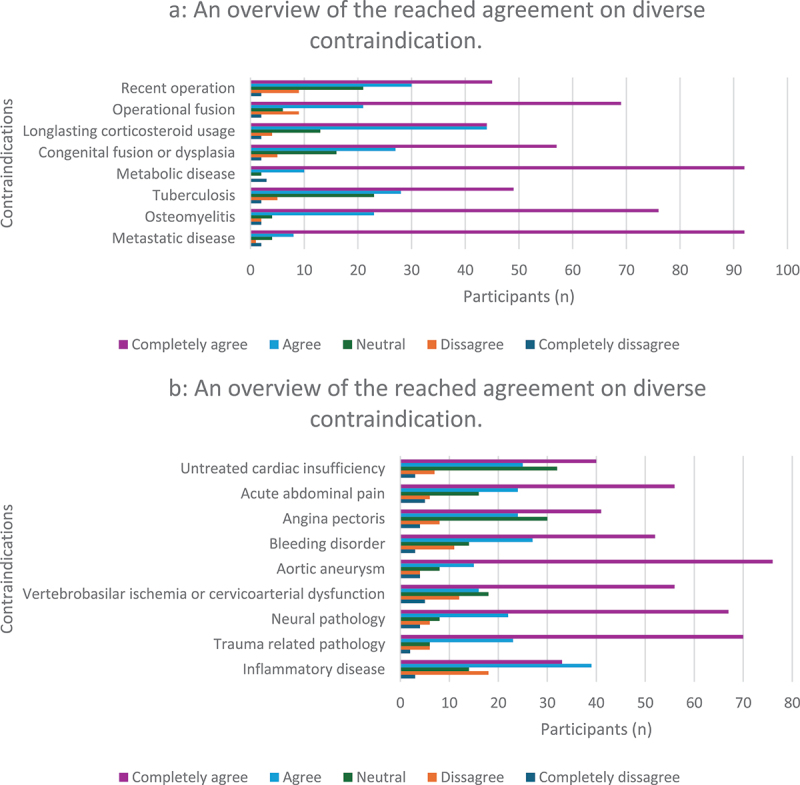
An overview of the reached agreement on diverse contraindication.

Most of the participants (36%) performed only a few TM per week, with an average frequency of 3–5 times per week. The majority (79%) indicated that they did not use any pre-screening tools prior to performing TM, but rather relied on their clinical expertise and examination. Moreover, most focused on the exclusion of red flags and the identification of articular dysfunctions found during examination as important supporting information. Nearly all participants stated that metabolic bone disease (*n* = 92) and metastatic disease (*n* = 92) were absolute contraindications (Figure 2(a, b)).

**Figure 3.**
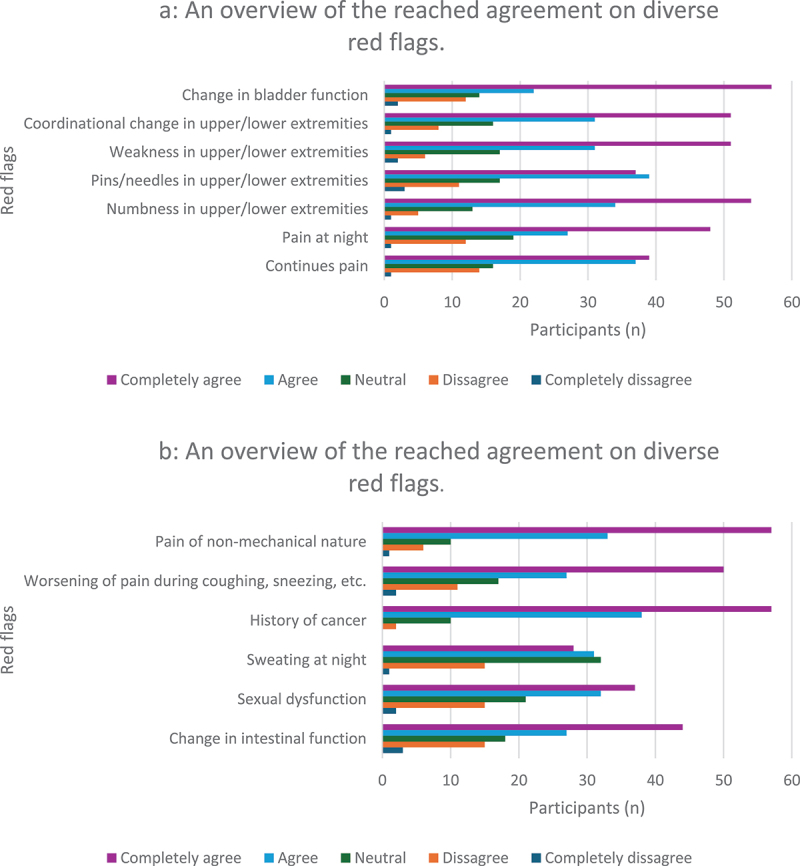
An overview of the reached agreement on diverse red flags.

When asked if there were any contraindications not previously listed, participants mentioned factors such as pregnancy, muscular spasms, and fear of manipulation. The main considerations before performing a TM were: 1) lack of symptom change after multiple TMs (*n* = 58); 2) osteopenia (*n* = 57); and 3) spondylolisthesis (*n* = 55). As for red flags (Figure 3(a, b)), the following top three were identified: 1) change in bladder function (*n* = 57), history of cancer (*n* = 57), and pain of non-mechanical nature (*n* = 57); 2) numbness in the upper/lower extremities or trunk (*n* = 54); and 3) weakness in the upper/lower extremities or trunk (*n* = 51), and change in coordination of upper/lower extremities (*n* = 51).

**Figure 4.**
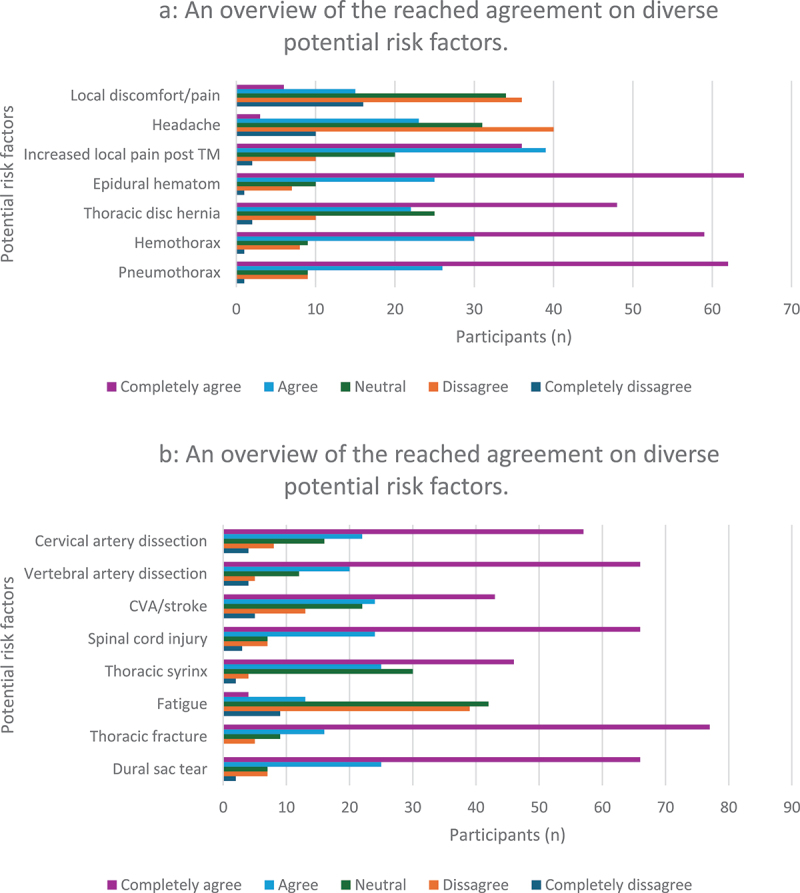
An overview of the reached agreement on diverse potential risk factors.

For potential risk factors (Figure 4(a, b)), the most frequent reported were: 1) thoracic fracture (*n* = 77); 2) dural sac tear (*n* = 66), spinal cord injury (*n* = 66), dissection of the vertebral artery (*n* = 66); and 3) epidural hematoma (*n* = 64). Nearly half (44%) did not differentiate between the upper and lower thoracic regions, followed by those who did (40%), with the remaining 16% being unsure. The majority found TM useful for dysfunctions in the thoracic spine (99%), but also the cervical spine (94%), ribs (91%), shoulders (90%), and lumbar spine (78%). Most therapists (*n* = 63) used TM to obtain biomechanical effects, followed by neurophysiological effects (*n* = 52), with the majority mentioning placebo as the least favored outcome (*n* = 71). TM was most preferred in the supine position (*n* = 44), followed by execution in the sitting position (*n* = 42). The prone posterior-to-anterior thrust technique scored the lowest, with 43 therapists reporting it as the least used. Despite the answers being preference-based, some described the supine position as more specific, whereas the sitting position was seen as more general and safer. A few mentioned the prone position as more dangerous due to a higher risk of costal complications. However, most agreed on the importance of using a technique that is comfortable for both therapist and patient.

### Treatment and intervention type

Regarding the interventions for managing thoracic dysfunctions, the most commonly mentioned were manipulations (98%), mobilizations (95%), and strengthening exercises (92%). On the topic of thoracic interventions as treatments for dysfunctions in other regions, almost all respondents referred to the cervical spine (95%), followed by the shoulder (90%) and the lumbar spine (79%). In cases of cervical spine pain, the most frequently used interventions were manipulations (93%), postural exercises (91%), and mobilizations (87%). For lumbar spine pain, therapists highlighted postural and strengthening exercises (85%), manipulation and mobilization (83%), and stretching (82%). Popular interventions for shoulder pain included postural exercises (84%); strengthening exercises, manipulations, and mobilizations (83%); and stretching (76%). As for elbow pain, mobilizations (52%), manipulations and postural exercises (51%), and strengthening exercises (50%) were preferred.

## Discussion

Most therapists rely on clinical expertise and examination for TM screening; however, substantial variability exists in the identification of contraindications, red flags, and risk factors, highlighting the need for a standardized consensus to support clinical decision-making.

### Demographics

A total of 107 participants were included, with a nearly equal distribution between genders. The group appeared relatively young, with most participants having received their physiotherapy and manual therapy licenses less than 10 years ago, approximately 10 years earlier than respondents in the previous survey [12]. Similar trends were observed in work setting, with nearly all therapists working in private practice. Despite the difference in participant numbers between the two surveys, it remains clear that most (manual) therapists in the current survey work in private practice: 94% compared to only 50% in the earlier study. Notable differences were observed in terms of obtained degrees. Manual therapy education in Belgium has significantly evolved over the years and continues to vary between universities. This is an important consideration, as it may influence clinical practice, particularly among those who are less experienced or received their licenses many years ago and may not be familiar with recent advancements and literature. Almost half of the respondents reported receiving fewer than 4 hours of additional training in the thoracic spine. Most therapists indicated treating between 6 and 9 patients with thoracic dysfunctions per week, which aligns with the findings from the previous survey, where the majority treated between 1 and 10 patients weekly.

### Thoracic complaints

A variety of thoracic complaints was reported in our study. Although the ribs play an important role in the thoracic spine function, contributing to both stability and respiration [19–21], only a very small proportion of the reported complaints were rib-related problems. As a result, rib-related issues were the third least frequently encountered thoracic complaint in clinical practice. Regarding the additional questioning of patients presenting with thoracic symptoms, a strong focus ( > 90%) was placed on respiratory function, such as pain during deep breathing and coughing or sneezing. Other commonly addressed topics included a history of cancer ( > 80%) and pain associated with posture or physical activity ( > 70%). It is worth noting the limited evidence supporting a clear link between posture and thoracic pain. For instance, some studies suggest that sagittal posture is not directly associated with pain [22]. Furthermore, one of the studies explored the relationship between low back pain and sitting behavior in sedentary office workers and found that discomfort may be less about ‘incorrect’ posture and more about the duration of static postures, concluding that the absence of micro-movements and prolonged, uninterrupted sitting periods may play a more significant role in symptom development [23]. Therefore, an excessive focus on posture by clinicians may be counterproductive, potentially reinforcing unnecessary fears or maladaptive beliefs in patients. When comparing the responses between the two surveys, both breathing and a history of cancer were included among the specific questions [12]. However, fewer clinicians in the earlier survey emphasized these topics, instead focusing more on osteoporosis and red flags. Differences also emerged between the surveys regarding physical examination. In the current study, palpation and AROM were prioritized. It remains unclear whether functional assessment, rated highly in the present findings, was utilized in the previous study, as the authors categorized some answers as ‘not specified.’ Additionally, muscle testing and ULTT, which were sometimes used by participants in this survey, were neither mentioned nor clearly defined by clinicians in the earlier study. The cervical spine, shoulder, and lumbar spine were frequently cited in relation to thoracic spine assessment when treating complaints in other regions. This aligns with established evidence and represents a positive trend, suggesting that the surveyed clinicians apply evidence-based clinical reasoning in their practice [8,10,11].

### Thoracic manipulations

The survey revealed that most therapists performed a relatively low number of TMs weekly, with 36% using the technique fewer than 10 times per week. This is comparable to previous findings, where the majority (71%) reported performing TMs between 1 and 10 times per week [12]. Additionally, most respondents (79%) indicated that they did not utilize a specific pre-screening tool prior to performing TMs, instead relying heavily on their clinical expertise and physical examination. Upon analysis, the primary focus during screening appeared to be exclusion of red flags and the identification of clear indications, such as articular dysfunctions, through examination. Although presented differently, these results are consistent with those of the previous survey, where patient history and physical examination were similarly emphasized. It is noteworthy that, in this survey, participants were specifically asked about the use of standardized guidelines as part of their screening process. In light of that, the results suggest a positive trend: therapists are applying TMs with caution and responsibility. This is essential, as thorough screening is a prerequisite for safe clinical practice [3,15–17,24]. However, it is important to recognize that a lack of standardization can lead to potential errors, highlighting the need for caution when relying solely on clinical expertise. This survey gathered diverse information on contraindications, red flags, and potential risk factors associated with TM. Although consensus was not always achieved, both this and previous survey identified metastatic disease and neurological pathologies as critical considerations. Furthermore, some participants appeared uncertain about how to interpret the term ‘potential risk factors.’ This ambiguity was also acknowledged by the authors of the previous survey, who emphasized that TM should be avoided when there is a high risk of AEs [12]. Given the variety in risk levels, futurer research should aim to establish clear distinctions, thereby supporting clinicians in making informed decision. Interestingly, both surveys showed nearly equal percentages of therapists (39% vs. 40%) reporting that they differentiate between the upper and lower thoracic spine during screening. These findings highlight a significant lack of literature regarding safe use of TM, especially when compared to the cervical and lumbar regions [15–17]. While some studies have offered valuable insights, additional research is necessary to draw firm conclusions and develop a potential clinical framework for TM [3,12–14]. Content analysis revealed that TM is also applied in managing dysfunctions in other regions, echoing previous survey results. Respondents commonly mentioned using TM for the cervical spine (94%), ribs (91%), shoulders (90%), and lumbar spine (78%). However, a notable shift in clinical reasoning was absevered: whereas most previous participants emphasized neurophysiological effects (52%), the majority in the current survey cited biomechanical effects (59%) as the primary rationale for using TM [12]. Similarly, in both surveys, clinicians reported placing the least emphasis on placebo effects. However, it is important to highlight that a significant number of therapists in both studies continue to focus on biomechanical explanations, despite several studies demonstrating limited support and even disproving a specific biomechanical mechanism [25]. Misinformation of this kind should not be taken lightly; clinicians must strive to deliver evidence-based care and ensure that their clinical reasoning remains accurate and up to date. Regarding technique preferences, supine positioning was favored, consistent with previous findings, with most clinicians prioritizing personal familiarity and patient comfort. Some participants described the supine position as being more specific and less dangerous compared to the prone posterior-to-anterior thrust. While the idea of a ‘specific’ manipulation may seem desirable, it is important to consider that the clinical effects of manipulation are largely attributed to neurophysiological responses rather than precise biomechanical targeting [25]. Therefore, the value of being ‘more specific’ in this context may be overstated. Similarly, categorizing a technique as more or less dangerous is difficult when preference and familiarity are mentioned. Clinicians should prioritize contraindications, red flags, and risk factors above personal technique preferences. That said, some caution regarding the prone posterior-to-anterior thrust is reasonable, as it may exert more compression on the ribcage. Nevertheless, it is unlikely that TM produces sufficient force to cause injury. According to previously published injury risk curves, a 10% risk of a rib fracture required 20% chest compression which corresponds to 4810N approximately whereas the maximal peak force obtained in the more recent study only reached 1167N [26]. Despite the overall low risk, it remains prudent to consider that increased force may elevate the likelihood of injury, especially when factoring in patient-specific characteristics such as age, degenerative changes, and sex.

### Treatment and intervention type

Interest was shown in the treatment and intervention types used for managing dysfunctions in the thoracic spine as well as in other regions. For the thoracic spine, the most commonly mentioned interventions were manual therapy techniques (namely spinal manipulations, mobilizations, and soft tissue massages), followed by exercises targeting posture, strength, and flexibility. Interestingly, thoracic spine interventions were also applied to treat dysfunctions in other anatomical regions. In addition to commonly associated areas such as the cervical spine or shoulder, less typical regions like the elbow were also mentioned. Although this line of reasoning is relatively uncommon, it may still be valid. Some evidence has demonstrated beneficial effects on elbow function, particularly in terms of pain-free grip after spinal mobilization and manipulation [27,28]. This may be partly explained by the regional interdependence theory, which proposes that impairments in remote anatomical areas that are unrelated to all appearances, still may contribute to or be associated with the primary complaint of a patient [29,30]. Interestingly, only 1% of participants considered thoracic spine interventions as a treatment option for costal complaints. Although perhaps not as commonly addressed as other regions, it is important to give careful consideration to patients presenting with thoracic complaints, particularly in the context of trauma. For instance, a Dutch national study found that approximately 6% of adult trauma patients sustained one or more rib fractures, and notably, 63% of those with rib fractures had thoracic trauma [31]. Additional interest was expressed regarding the types of interventions used in patients with pain in the cervical and lumbar spine, shoulder, and elbow. In general, similar patterns emerged across regions, with most participants reporting the use of: 1) manipulations; 2) exercises; and 3) mobilizations. Nonetheless, a few other interventions were noted. For the lumbar spine specifically, taping (21%) and acupuncture (19%) were mentioned. Taping, often referring to kinesio tape (KT), is a commonly used technique; however, its efficacy remains questionable. For example, a systematic review with meta-analysis found no evidence supporting the use of KT in clinical practice for patients with chronic nonspecific low back pain [32]. In the context of our study, no definitive conclusions could be drawn regarding taping, as participants did not specify the type of tape used. Acupuncture, on the other hand, has shown some promise for lumber spine treatment [33]. Nonetheless, it is important to consider the need for further research as a lack of consensus can be found in other studies [34,35].

### Strengths and limitations

The current survey aimed to build upon the previous study [12] incorporating recent insights alongside clinical expertise. However, the anonymity of the survey presented certain limitations. For example, due to the inability to contact participants for clarification on certain responses, particularly those related to educational background, these had to be excluded from analysis. Open-ended questions were included to gain a deeper understanding of the clinical reasoning behind participants’ responses. However, this may have contributed to a lower response rate [36]. The internal consistency of different dimensions was not assessed as the goal was to map a broad spectrum of diagnostic classifications and symptoms. Additionally, the survey was conducted in Dutch, which unintentionally excluded therapists from the French-speaking region of Belgium. Nevertheless, given the standardized IFOMPT-accredited manual therapy education across Belgium, no significant differences in clinical practice are expected between the two language communities. All questions were mandatory, which may have reduced motivation to complete the survey. However, this ensured that only fully completed surveys were collected. Lastly, the likelihood of multiple submissions was minimized due to e-mail recognition.

## Implications for clinical practice and future research

This study offers valuable insights into the knowledge and use of TM by manual therapists in Belgium and the Netherlands. The findings emphasize the critical role of sound clinical reasoning in guiding interventions such as TM. While a certain degree of consensus was observed among participants, notable variability in responses highlights the current lack of clarity surrounding the thoracic spine, especially when compared to the more extensively studied cervical and lumbar regions. Despite this variation, the data gathered contributes meaningfully to clinical reasoning and provides an informative overview of TM practices. Future research should focus on developing a standardized screening framework specific to the thoracic spine. Such a framework would enhance practitioner confidence, offer clear guidance, and help reduce the risk of AEs associated with TM.

## Conclusion

This study found that while most manual therapists in Belgium and the Netherlands do not utilize a standardized pre-screening tool, all reported relying heavily on the exclusion of red flags and the identification of clear indications during examination. Despite some encouraging overlap and areas of consensus, considerable variation was observed in the interpretation of contraindications, red flags, and potential risk factors. Additionally, although a large proportion of therapists do not differentiate between the upper and lower thoracic spine, the near-equal distribution of responses indicates a lack of clarity on this matter. Many therapists reported using TM not only for thoracic dysfunctions but also for conditions in other anatomical regions. When performing TM, clinicians most commonly aimed for biomechanical effects, with the supine position being the preferred technique.

## References

[1] Evans DW, Lucas N. What is manipulation? A new definition. BMC Musculoskelet DisorD. 2023;24(1):194. doi: 10.1186/s12891-023-06298-w 36918833 PMC10015914

[2] Gyer G, Michael J, Inklebarger J, et al. Spinal manipulation therapy: is it all about the brain? A current review of the neurophysiological effects of manipulation. J Integr Med. 2019;17(5):328–337. doi: 10.1016/j.joim.2019.05.004 31105036

[3] Puentedura EJ, O’Grady WH. Safety of thrust joint manipulation in the thoracic spine: a systematic review. J Man Manipulative Ther. 2015;23(3):154–161. doi: 10.1179/2042618615Y.0000000012 PMC4534851 26309386

[4] Heneghan NR, Webb K, Mahoney T, et al. Thoracic spine mobility, an essential link in upper limb kinetic chains in athletes: a systematic review. Transl Sports Med. 2019;(6):1–15. doi: 10.1002/tsm2.109

[5] Tsang SMH, Szeto GPY, Lee RYW. Normal kinematics of the neck: the interplay between the cervical and thoracic spines. Man Ther. 2013;18(5):431–437. doi: 10.1016/j.math.2013.03.002 23632368

[6] Casanova-Mendez A, Oliva-Pascual-Vaca A, Rodriguez-Blanco C, et al. Comparative short-term effects of two thoracic spinal manipulation techniques in subjects with chronic mechanical neck pain: a randomized controlled trial. Man Ther. 2014;19(4):331–337. doi: 10.1016/j.math.2014.03.002 24679838

[7] Cleland JA, Childs MJD, McRae M, et al. Immediate effects of thoracic manipulation in patients with neck pain: a randomized clinical trial. Man Ther. 2005;10(2):127–135. doi: 10.1016/j.math.2004.08.005 15922233

[8] Masaracchio M, Kirker K, States R, et al. Thoracic spine manipulation for the management of mechanical neck pain: a systematic review and meta-analysis. PLOS ONE. 2019;14(2):e0211877. doi: 10.1371/journal.pone.0211877 30759118 PMC6373960

[9] Muth S, Barbe MF, Lauer R, et al. The effect of thoracic spine manipulation in subjects with signs of rotator cuff tendinopathy. J Orthopaedic Sports Phys Ther. 2012;42(12):1005–1016. doi: 10.2519/jospt.2012.4142 22951537

[10] Peek AL, Miller C, Heneghan NR. Thoracic manual therapy in the management of non-specific shoulder pain: a systematic review. J Man Manipulative Ther. 2015;23(4):176–187. doi: 10.1179/2042618615Y.0000000003 PMC4727730 26917935

[11] Sung YB, Lee JH, Park YH. Effects of thoracic mobilization and manipulation on function and mental state in chronic lower back pain. The J Phys Ther Sci. 2014;26(11):1711–1714. doi: 10.1589/jpts.26.1711 25435683 PMC4242938

[12] Heneghan NR, Puentedura EJ, Arranz I, et al. Thoracic thrust joint manipulation: an international survey of current practice and knowledge in IFOMPT member countries. Musculoskeletal Sci Pract. 2020;50:102251. doi: 10.1016/j.msksp.2020.102251 32992076

[13] Heneghan NR, Davies SE, Puentedura EJ, et al. Knowledge and pre-thoracic spinal thrust manipulation examination: a survey of current practice in the UK. J Man Manipulative Ther. 2018;26(5):301–309. doi: 10.1080/10669817.2018.1507269 PMC6237157 30455557

[14] Puentedura EJ, Slaughter R, Reilly S, et al. Thrust joint manipulation utilization by U.S. physical therapists. J Man Manipulative Ther. 2017;25(2):74–82. doi: 10.1080/10669817.2016.1187902 PMC5430452 28559666

[15] Rushton A, Carlesso L, Flynn T, et al. International framework for examination of the cervical region for potential of vascular pathologies of the neck prior to orthopaedic manual therapy (OMT) intervention: international IFOMPT cervical framework. 2020:1–42. https://www.ifompt.org/site/ifompt/IFOMPT%20cervical%20framework%20final%202020%20Add%202023.pdf

[16] DePalma MG. Red flags of low back pain. Am Acad Physician Assistants. 2020;33(8):8–11. doi: 10.1097/01.JAA.0000684112.91641.4c 32740106

[17] Koninklijk Nederlands Genootschap voor Fysiotherapie (KNGF); Vereniging van Oefentherapeuten Cesar en Mensendieck (VvOCM). KNGF-richtlijn Lage rugpijn en lumbosacraal radiculair syndroom. Amersfoort/Utrecht: KNGF/VvOCM. 2021. Available from: https://www.kennisplatformfysiotherapie.nl/app/uploads/sites/2/2024/11/kngf_richtlijn_lage_rugpijn_en_lrs_2021.pdf.

[18] Sharma A, Minh NT, Thang TLL, et al. A consensus-based checklist for reporting of survey studies (CROSS). J Gen Intern Med. 2021;36(10):3179–3187. doi: 10.1007/s11606-021-06737-1 33886027 PMC8481359

[19] Beyer B, Van Sint Jan S, Chèze L, et al. Relationship between costovertebral joint kinematics and lung volume in supine humans. Respir Physiol Neurobiol. 2016;232:57–65. doi: 10.1016/j.resp.2016.07.003 27421681

[20] Liebsch C, Wilke HJ. Rib presence, anterior rib cage integrity and segmental length affect the stability of the human thoracic spine: an in vitro study. Front Bioeng biotechnol. 2020;8(46). doi: 10.3389/fbioe.2020.00046 PMC7018667 32117927

[21] Lin Y, Tan H, Rong T, et al. Impact of thoracic cage dimension and geometry on cardiopulmonary function in patients with congenital scoliosis. The Spine J. 2019;44(20):1441–1448. doi: 10.1097/BRS.0000000000003178 31365514

[22] Christensen ST, Hartvigsen J. Spinal curves and health: a systematic critical review of the epidemiological literature dealing with associations between sagittal spinal curves and health. J Manipulative Physiol Ther. 2008;31(9):690–714. doi: 10.1016/j.jmpt.2008.10.004 19028253

[23] Bontrup C, Taylor WR, Fliesser M, et al. Low back pain and its relationship with sitting behaviour among sedentary office workers. Appl Ergon. 2019;81:102894. doi: 10.1016/j.apergo.2019.102894 31422243

[24] Rushton A, Rivett D, Carlesso L, et al. International framework for examination of the cervical region for potential of cervical arterial dysfunction prior to orthopaedic manual therapy intervention. Man Ther. 2014;19(3):222–228. doi: 10.1016/j.math.2013.11.005 24378471

[25] Bialosky JE, Beneciuk JM, Bishop MD, et al. Unraveling the mechanisms of manual therapy: modeling an approach. J Orthop Sports Phys Ther. 2018;48(1):8–18. doi: 10.2519/jospt.2018.7476 29034802

[26] Beyer B, Michaud A, Oliver T, et al. Investigation of reaction force magnitude and orientation during supine thoracic thrust manipulation applied to intervertebral and costovertebral regions. Musculoskeletal Sci Pract. 2020;49:102217. doi: 10.1016/j.msksp.2020.102217 32861370

[27] Fernandez-Carnero J, Cleland JA, Arbizu RLT. Examination of motor and hypoalgesic effects of cervical vs thoracic spine manipulation in patients with lateral epicondylalgia: a clinical trial. J Manipulative Physiol Ther. 2011;34(7):432–440. doi: 10.1016/j.jmpt.2011.05.019 21875517

[28] Zunke P, Auffarth A, Hitzi W, et al. The effect of manual therapy to the thoracic spine on pain-free grip and sympathetic activity in patients with lateral epicondylalgia humeri. A randomized, sample sized planned, placebo-controlled, patient-blinded monocentric trial. BMC Musculoskelet DisorD. 2020;21(1):186. doi: 10.1186/s12891-020-3175-y 32209068 PMC7093973

[29] McDevitt A, Young J, Mintken P, et al. Regional interdependence and manual therapy directed at the thoracic spine. J Man Manipulative Ther. 2015;23(3):139–146. doi: 10.1179/2042618615Y.0000000005 PMC4534849 26309384

[30] Wainner RS, Whitman JM, Cleland JA, et al. Regional interdependence: a musculoskeletal examination model whose time has come. J Orthopaedic Sports Phys Ther. 2007;37(11):658–660. doi: 10.2519/jospt.2007.0110 18057674

[31] Elkins MR. Physiotherapy management of rib fractures. J physiother. 2023;69(4):211–219. doi: 10.1016/j.jphys.2023.08.016 37714770

[32] Júnior MADL, Almeida MOD, Santos RS, et al. Effectiveness of kinesio taping in patients with chronic nonspecific low back pain: a systematic review with meta-analysis. Spine (phila Pa 1976). 2019;44(1):68–78. doi: 10.1097/BRS.0000000000002756 29952880

[33] Oka H, Matsudaira K, Takano Y, et al. A comparative study of three conservative treatments in patients with lumbar spinal stenosis: lumbar spinal stenosis with acupuncture and physical therapy study (LAP study). BMC Complement Altern Med. 2018;18(1):19. doi: 10.1186/s12906-018-2087-y 29351748 PMC5775532

[34] Luo D, Liu Y, Wu Y, et al. Warm needle acupuncture in primary osteoporosis management: a systematic review and meta-analysis. Acupuncture Med. 2018;36(4):215–221. doi: 10.1136/acupmed-2016-011227 PMC6089200 29986901

[35] Qin Z, Ding Y, Xu C, et al. Acupuncture vs noninsertive sham acupuncture in ageing patients with degenerative lumbar spinal stenosis: a randomized controlled trial. Am J Med. 2019;133(4):500–507.e20. doi: 10.1016/j.amjmed.2019.08.038 31525334

[36] VanGeest JB, Johnson TP, Welch VL. Methodologies for improving response rates in surveys of physicians: a systematic review. Evaluation Health Professions. 2007;30(4):303–321. doi: 10.1177/0163278707307899 17986667

